# Mesoscopic Modeling of Fracture in Heterogeneous Bituminous Polymer Composites: Coupling Random Aggregate Distribution with Bilinear Cohesive Zone Models

**DOI:** 10.3390/polym18091139

**Published:** 2026-05-06

**Authors:** Wenjing Li, Hang Gao, Linyu Xie, Zhifei Tan, Peng Cao

**Affiliations:** 1 College of Architecture and Civil Engineering, Beijing University of Technology, Beijing 100124, China; wenjingli1022@126.com (W.L.); xielinyu2023@126.com (L.X.); zhifeitan@bjut.edu.cn (Z.T.); 2 The Institute of Xi’an Aerospace Solid Propulsion Technology, Xi’an 710025, China; 18829230700@163.com

**Keywords:** polymer-matrix composites, viscoelasticity, fracture behavior, cohesive zone model, mesoscopic modeling

## Abstract

The fracture of bituminous polymer composites is fundamentally dictated by microstructural heterogeneity and the complex viscoelasticity of the asphalt matrix. This study develops a robust numerical framework coupling a random polygonal aggregate distribution algorithm with a bilinear cohesive zone model (CZM) to simulate fracture mechanics in heterogeneous asphalt-based composites. A key feature of the model is the explicit accounting for the stochastic distribution of the coarse aggregate and the time-dependent mechanical response of the fine aggregate matrix (FAM). Following experimental validation via frequency sweep and semi-circular bending (SCB) tests, a multi-scale parametric analysis was conducted to quantify the impacts of aggregate gradation, volume fraction, and shape. Results demonstrate that mixtures with high percentages of large-sized aggregates effectively delay macroscopic fracture by increasing the energy dissipation required for cracks to bypass the aggregate phase. While increasing the volume fraction of aggregates improves peak strength, it simultaneously accelerates post-peak load deterioration and reduces total fracture work, indicating a critical loss in the composite’s deformation capacity. Furthermore, particles with higher angularity provide superior blocking effects compared to rounded counterparts. This research offers a high-efficiency computational tool for the structural optimization of highly filled composites and provides critical insights into their internal stress states and macroscopic fracture mechanics.

## 1. Introduction

Asphalt concrete (AC) is a complex, multi-phase polymer composite widely utilized in global pavement infrastructure [1,2,3,4,5,6]. Traditional macroscopic continuum models typically treat AC as a homogeneous material, often overlooking the influence of its intricate internal structure. This limitation hinders the accurate prediction of discrete crack initiation and the complex propagation paths that occur at the mesoscopic scale. 

To address these challenges, microstructural modeling has emerged as a robust approach for investigating the fracture behavior of these polymer-based composites [7]. By explicitly representing the physical interactions among constituent phases—specifically the coarse aggregate particles, the fine aggregate matrix (FAM), and air voids—mesoscopic models provide a more realistic depiction of the material’s failure mechanisms [8,9,10,11,12]. In this composite system, aggregates constitute approximately 70% of the volume, serving as the primary structural skeleton [13,14]. 

The morphological properties of these aggregates, including their size distribution (gradation), volume content, and angular shape, fundamentally dictate the load-bearing capacity and cracking resistance of the composite [15,16,17]. Research has demonstrated that coarser gradations enhance fracture toughness by facilitating particle interlocking [18,19]. Furthermore, the specific geometry and angularity of these aggregate particles significantly influence the crack trajectory; rather than propagating linearly, cracks are forced to deflect and meander to bypass the harder particles [18,20]. This crack deflection mechanism requires a significantly greater dissipation of fracture energy, thereby enhancing the overall resistance of the polymer mixture [18,21,22]. 

Accurate reconstruction of the AC microstructure is critical to simulating these mesoscopic interactions [23,24]. Currently, Digital Image Processing (DIP) and random aggregate generation algorithms are the two principal techniques employed [25,26,27,28]. While DIP [29] provides an exact digital replica, its high computational cost and reliance on advanced scanning equipment often restrict its application for large-scale parametric studies [8,30]. Alternatively, random aggregate packing [29] algorithms offer a highly efficient method to generate statistically equivalent microstructures, allowing researchers to systematically isolate the influence of specific design factors [27,31]. By representing aggregate particles as randomly distributed polygons, researchers can systematically isolate and evaluate the influence of specific design factors—such as aggregate shape and volume—across a broad range of simulated scenarios [32,33].

Once the heterogeneous microstructure is established, an appropriate fracture model must be applied. Traditional linear elastic fracture mechanics (LEFM) is often inadequate for AC due to the presence of a relatively large fracture process zone (FPZ) ahead of the crack tip [34]. To overcome this stress singularity issue, the cohesive zone model (CZM) provides a superior phenomenological framework [35,36,37]. By embedding cohesive elements along potential crack paths, the CZM can successfully capture spontaneous crack nucleation, branching, and propagation [34,37]. Furthermore, because the FAM exhibits highly time- and rate-dependent properties, coupling the CZM with the viscoelastic characteristics of the matrix is essential for accurately simulating the quasi-brittle fracture behavior of AC [27,35,38,39].

Despite the recognized influence of material heterogeneity, there remains a lack of comprehensive computational studies that systematically isolate the coupled effects of aggregate gradation, volume fraction, and specific polygonal shapes on AC fracture mechanisms. To address this gap, this study develops a coupled mesoscopic finite element (FE) framework utilizing a random polygonal aggregate distribution algorithm and a viscoelastic bilinear CZM. The semicircular bending (SCB) test is simulated to evaluate fracture performance due to its high repeatability. The primary objective of this research is to quantitatively model how aggregate morphology and distribution alter crack trajectories and energy dissipation, ultimately providing theoretical guidance for optimizing the microstructural design of anti-cracking pavement. To achieve this, the following tasks were conducted: (1) conduct frequency sweep tests on FAM to capture the viscoelastic properties of the asphalt matrix; (2) perform laboratory SCB tests to obtain the macroscopic fracture properties of the AC; (3) develop 2D microstructural SCB models using a random polygonal aggregate distribution program and insert cohesive elements; (4) calibrate and verify the developed numerical models via inverse calibration against experimental SCB results; and (5) systematically evaluate the distinct and coupled effects of aggregate shape (focusing on crack deflection mechanisms), gradation, and volume content on the overall fracture performance.

## 2. Theory on Viscoelastic Models and Cohesive Zone Modeling

### 2.1. Linear Viscoelastic Characterization

The mechanical response of the FAM is significantly influenced by its inherent viscoelasticity, which dictates the internal stress states of the AC under varying loading rates. To capture this behavior, the viscoelastic properties measured via frequency sweep tests are described using a master curve. The time–temperature superposition principle, based on the Williams–Landel–Ferry (WLF) formulation [40], was applied to shift the experimental data at various temperatures to a reference temperature of 10 °C. The shifted data were then fitted using the modified Huet–Sayegh (MHS) model, which accurately captures the complex modulus of the asphalt binder and mixture over a wide frequency range [41,42,43].

For implementation within the FE framework, the analytical MHS master curves were converted into a Prony series (generalized Maxwell) model. This conversion involves determining the relaxation moduli and relaxation times by minimizing the residuals between the storage and loss moduli of the analytical and Prony-based models. The time-domain relaxation modulus, E(t), is mathematically expressed as follows:

(1) $$E(t)=E_{\infty }+\sum_{i=1}^{n}E_{i}e^{-t/\tau_{i}}.$$

where $E_{\infty }$ is the long-term equilibrium modulus, $E_{i}$ represents the relaxation modulus of the i-th Maxwell branch, $\tau_{i}$ is the corresponding relaxation time, and n is the number of terms in the series. 

Through Fourier transform, the time-domain Prony series terms can be converted to the frequency domain Prony series terms using the following equations:

(2) $$E^{\prime }(\omega )=E_{0}\left(1-\sum_{i=1}^{n}g_{i}\frac{{\left(\omega \tau_{i}\right)}^{2}}{1+{\left(\omega \tau_{i}\right)}^{2}}\right)$$

(3) $$E^{\prime \prime }(\omega )=E_{0}\sum_{i=1}^{n}g_{i}\frac{\omega \tau_{i}}{1+{\left(\omega \tau_{i}\right)}^{2}}$$

where $E^{\prime }$ is the storage modulus, $E^{\prime \prime }$ is the loss modulus, ω is the angular frequency, $E_{0}$ is the instantaneous modulus; and $g_{i}=E_{i}/E_{0}$ defines the weight of the ith Maxwell element.

The Prony series parameters are determined by performing a regression analysis based on laboratory measurements. First, master curves for the storage and loss moduli are constructed from DSR tests (detailed in Section 3). The optimal Prony series coefficients are then identified by minimizing the error between these experimental values and the predicted values derived from the Prony series model (Equations (2) and (3)) [9,10]. This is achieved by minimizing the following objective function:

(4) $$\mathrm{Objective}\;\mathrm{function}=\sum_{i=1}^{m}\left({\left(\frac{E^{\prime }(\omega_{i}{)}_{pred}}{E^{\prime }(\omega_{i}{)}_{cal}}-1\right)}^{2}+{\left(\frac{E^{\prime \prime }(\omega_{i}{)}_{pred}}{E^{\prime \prime }(\omega_{i}{)}_{cal}}-1\right)}^{2}\right)$$

where m is the total data points used; $E^{\prime }(\omega_{i}{)}_{cal}$ and $E^{\prime \prime }(\omega_{i}{)}_{cal}$ represent the calculated storage and loss modulus at the ith frequency based on the constructed master curves, respectively; and $E^{\prime }(\omega_{i}{)}_{pred}$ and $E^{\prime \prime }(\omega_{i}{)}_{pred}$ are the predicted storage and loss modulus at the ith frequency based on the Prony series model parameters, respectively. 

### 2.2. Cohesive Zone Model

**Figure 1:**
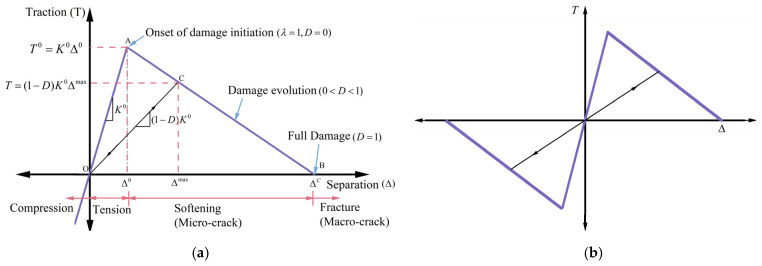
Schematic of bilinear CZM: (**a**) normal component; (**b**) tangential component [45].

As shown in Figure 1, a bilinear CZM was implemented to simulate crack propagation in the FAM. The traction-separation law controls the constitutive response of the cohesive element. At the first stage, the traction (T) linearly increases with the separation (Δ) until reaching the maximum value, the cohesive strength ($T^{0}$). When the traction reaches the cohesive strength, the damage is activated. In this study, the onset and development of damage in the FAM were defined by the maximum stress criterion. The damage will be triggered when each of the three maximum stresses, i.e., stresses in the normal direction ($T_{1}$), in the first tangential direction ($T_{2}$), and in the second tangential direction ($T_{3}$), reaches its maximum value, which can be expressed as follows:

(5) $$\lambda =MAX\left\{\frac{T_{1}}{T_{1}^{0}},\frac{T_{2}}{T_{2}^{0}},\frac{T_{3}}{T_{3}^{0}}\right\}=1$$

When the damage is triggered, the stiffness of the cohesive element linearly decreases with the separation. This is described by the stiffness degradation parameter (D), which ranges from 0 to 1. When equal to 1, the cohesive element reaches its effective separation ($\Delta^{c}$) and T equals to zero. D and T in the whole process can be calculated by the following equations:

(6) $$T=(1-D)K^{0}\Delta$$

(7) $$D=\frac{\Delta_{c}(\Delta^{\max}-\Delta^{0})}{\Delta^{\max}(\Delta^{c}-\Delta^{0})}$$

where $\Delta^{c}$ is the effective separation at complete failure; $\Delta^{\max}$ is the maximum effective separation during the loading history; $\Delta^{0}$ is the effective separation at damage initiation; $K^{0}$ represents the initial elastic stiffness of the cohesive interface. This parameter defines the material’s resistance to interfacial separation prior to the initiation of micro-cracking or damage, corresponding to the slope of the initial linear-elastic ascending branch of the traction-separation curve (as illustrated in Figure 1). 

The whole process of damage evolution is described by the dissipated fracture work, which is equal to the area under the traction–separation curve as shown in Figure 1. Therefore, it can be expressed by the cohesive strength ($T^{0}$), and the separation (Δ) as shown in the following equation [44]:

(8) $$G=\int_{0}^{\Delta }T(\Delta )d\Delta =\frac{1}{2}T^{0}\Delta$$

While Figure 1 illustrates the theoretical bilinear traction-separation law governing individual micro-interfaces, the reliability and physical applicability of this cohesive zone model are validated at the macroscopic structural scale. As detailed subsequently in Section 4, when this theoretical CZM is applied to the thousands of interfacial elements within the heterogeneous AC model, their collective micro-mechanical energy dissipation accurately replicates the global macroscopic force–displacement response observed in the physical laboratory SCB tests. 

## 3. Materials and Laboratory Tests

The material selection and experimental program were designed to characterize the constituent properties of the asphalt mixture and provide the necessary data for numerical model validation. A dense-graded wearing course mixture (WC10) with a 10 mm nominal maximum aggregate size was selected for laboratory characterization.

For the purpose of microstructural modeling, the FAM was defined as the component consisting of aggregates smaller than 1.18 mm and the asphalt binder. The volumetric compositions of the FAM were calculated using the analytical method proposed by Underwood and Kim [46]. This approach assumes that all aggregate particles larger than 1.18 mm are coated by a mastic film approximately 29.5 um thick. The detailed gradation and the resulting mass and volume compositions for both the WC10 mixture and the FAM are summarized in Table 1.

**Figure 2:**
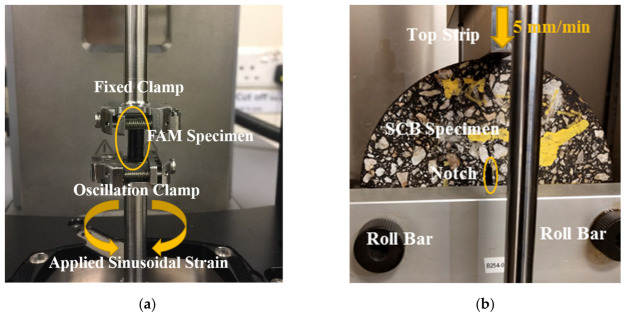
Laboratory tests: (**a**) FAM test; (**b**) AC SCB test.

To characterize the viscoelastic response of the asphalt matrix, frequency sweep tests were conducted on FAM specimens using a specialized geometry developed by Huurman et al. [47,48]. As shown in Figure 2a, an Anton Paar MCR702 dynamic shear rheometer (DSR) (Anton Paar GmbH, Graz, Austria) with a specific configuration was utilized to measure the complex moduli [49]. Testing was performed at temperatures of 0, 10, 20, 30, and 40 °C across a frequency range of 0.1 Hz to 100 Hz. The acquired experimental data were then employed to construct the master curve at a reference temperature of 10 °C via time–temperature superposition as detailed in Section 2.1.

The macroscopic fracture behavior of the heterogeneous asphalt mixture was evaluated through laboratory SCB tests. Test specimens were prepared by fabricating 150 mm-diameter AC samples using a Superpave gyratory compactor, which were then cut into semicircular specimens with a thickness of 50 mm. A 10 mm vertical notch was cut at the bottom center of each specimen to control crack initiation. As shown in Figure 2b, the SCB tests were performed at 10 °C using a universal testing machine (UTM) following the configuration specified in the BS EN 12697-44 standard [50]. A vertical load was applied at a constant rate of 50 mm/min until the specimen reached total fracture. The results from these laboratory tests served as the primary benchmark for calibrating the cohesive zone model and verifying the accuracy of the random aggregate distribution algorithm.

## 4. Model Development and Verification

### 4.1. 2D Microstructure Model Generation

**Figure 3:**
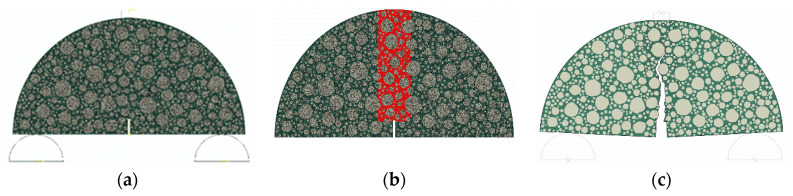
SCB microstructural model: (**a**) Sample configuration. (**b**) Cohesive element zone. (**c**) Cracked SCB sample.

In this study, a subroutine program based on the ABAQUS platform was developed to construct 2D microstructure models. This method allows for the generation of aggregates with variable angularity and concave–convex morphologies, ensuring the aggregate particles occupy the specimen space according to the specific target gradation of the mixture. By utilizing an optimized placement algorithm, the subroutine ensures that the numerical models maintain a volumetric composition and spatial distribution identical to the experimental specimens. Following the generation of the aggregate skeleton, the model is partitioned into distinct mechanical phases, specifically the coarse aggregate particles and the FAM. Through Boolean operations, the subroutine differentiates these phases to assign independent viscoelastic and linear elastic material properties. Figure 3a displays a generated SCB microstructure model. To model the fracture behavior, zero-thickness bilinear cohesive elements were batch-inserted into the mesh. These 4-node two-dimensional cohesive elements (COH2D4) were strategically placed both within the FAM to represent mastic tearing and along the aggregate-matrix interfaces to simulate interfacial debonding. To balance computational accuracy with efficiency, this high-density insertion was localized within the central 20 mm area of the SCB specimen as shown in Figure 3b, where the highest internal stress concentrations and crack propagation are statistically most likely to occur.

### 4.2. FE Simulation and Model Verification

The FE simulations were conducted to model the crack propagation of AC under vertical loading. As shown in Figure 3a, the two roll bars at the bottom were fixed on the center points, respectively. To ensure a low resistance of the roll bars to the SCB sample under loading, a low friction coefficient of 0.05 was assigned to the contact faces between the SCB sample and roll bars. A vertical load with a loading rate of 5 mm/min was applied at the center of the top strip to simulate the loading process in the laboratory test. Through the FE simulation, the displacement and reaction force on the top strip were recorded against time. Figure 3c displays the cracked SCB sample by simulation. The crack can bypass the aggregate particles and propagate through the matrix and along the surface of the aggregate particles. In this study, a 2016 version of the computer server with 10 cores of 2.8 GHz Intel^®^ Xeon^®^ Processors E5-2680 v2 was used, and it took approximately 40 min to complete one simulation of SCB loading for 35 s. 

In this model, the material properties of three phases including FAM, aggregate, and cohesive elements were determined. The aggregate particles were assumed to be linear elastic with an elastic modulus of 60 GPa and a Poisson’s ratio of 0.20. For the FAM, a viscoelastic constitutive model was applied utilizing a Poisson’s ratio of 0.4 and the Prony series formulation (Table 2). The specific Prony series parameters were determined through the regression analysis detailed in Section 3; it was found that a 12-term Prony series provides sufficient accuracy, yielding a relative error of less than 3% compared to the constructed master curves.

For the cohesive elements, the material properties were determined by utilizing experimental results and subsequently verified by statistical analysis from the modeling. Cohesive zone modeling provides a highly efficient phenomenological framework to remove stress singularities at the crack tip and accurately model localized failure [51]. Because the direct measurement of the mesoscopic fracture properties of the in situ FAM is highly complex, these parameters were determined through a rigorous, iterative inverse calibration process. 

Initial parameter estimates, based on the existing literature [52], were systematically adjusted within the numerical model. Specifically, an objective function was employed to minimize the least squares error between the simulated macroscopic load–displacement response and the laboratory SCB test data. During this iterative process, a clear physical mapping of the parameters was established: the cohesive strength primarily governs the peak macroscopic load required for crack initiation, while the total fracture work dictates the post-peak softening trajectory and the energy dissipation capacity of the mixture.

**Figure 4:**
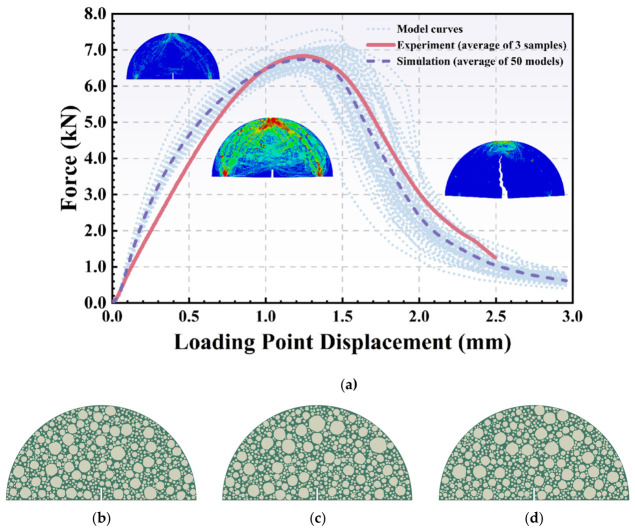
Generated models and predicted results based on the developed fifty models: (**a**) force–displacement curves of simulations and experimental results and (**b**–**d**) examples of generated models with varying aggregate particle spatial distribution.

By continuously optimizing these variables until the simulated global response closely matched the experimental data, the precise mesoscopic parameters were finalized. Consequently, a maximum stress of 3 MPa and a fracture work of 1200 J/m^2^ were defined for the cohesive elements, respectively. The average force–displacement curves from the laboratory tests and the fifty microstructural models utilizing these calibrated parameters are displayed in Figure 4a. It can be seen that the average force–displacement curve from the modeling shows high consistency with the experimental results, verifying the accuracy of the inverse calibration procedure.

Furthermore, while the 50 simulated curves demonstrate a stable average response, they inherently exhibit sample-to-sample variation. This variation is entirely attributable to the stochastic spatial distribution of the aggregate particles generated by the algorithm. Although all 50 models were constructed utilizing the exact same target gradation, volume fraction, and aggregate shape, the specific microstructural arrangement of the aggregate phase differs in every individual realization (as illustrated by the distinct spatial distributions shown in Figure 4b–d). Consequently, the propagating crack encounters unique localized stress concentrations and must navigate a differently tortuous path in each model. This varying crack trajectory directly leads to the observed statistical distribution in the predicted macroscopic force–displacement curves, accurately reflecting the inherent heterogeneity of actual asphalt concrete composites.

**Figure 5:**
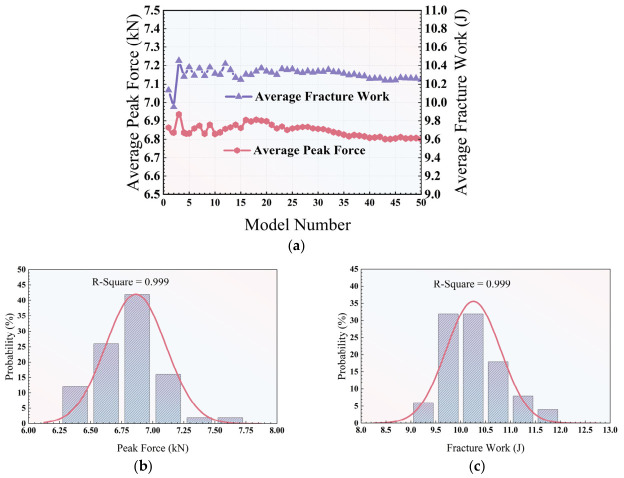
Statistical analysis of the results of fifty models: (**a**) Average peak force and average fracture work vs. model number. (**b**) The probability distribution of peak force. (**c**) The probability distribution of fracture work.

To evaluate the statistical performance of the simulation results, statistical analysis was performed on the simulated results of the fifty models. The peak force and total fracture work were selected as the two parameters in the statistical analysis. Figure 5 presents the statistical results of both parameters. From Figure 5a, it can be seen that fracture work shows larger variations than peak force, but the variations in both parameters decrease and reach stable values with the increasing number of models. In addition, to identify the probability distributions of peak force and fracture work, the probability distributions of both parameters are plotted as presented in Figure 5b,c. It can be observed that the normal distributions fit well with the probability distributions of peak force and fracture work by giving the high R square values of 0.999 for both parameters. Therefore, it can be concluded that the fifty simulations are large enough to ensure that both the peak force and fracture work obey the normal distributions.

To check whether the parameters of the cohesive element model are reliable, Student’s *t*-test, a widely used statistical method to compare the difference between the means of two groups, was further applied to statistically evaluate the reliability of the proposed model. Both the peak force and total fracture work were evaluated in Student’s *t*-test analysis. The null hypothesis is that the peak force (or the fracture work) from the model simulations is equal to that from the laboratory tests. According to Student’s *t*-test results presented in Table 3, this hypothesis is statistically true at a confidence level of 95%, indicating no significant difference between the simulation and experimental results. In other words, the statistical analysis verified the reliability of the proposed model and the assumed fracture parameters.

To apply the random aggregate distribution algorithm for analyzing the fracture behavior of AC, it is important to determine the appropriate number of simulations, because if the number is too small, the simulations cannot draw conclusions with statistical significance, while if it is too big, the computational cost will be too high. Therefore, the following equation was used to determine the minimum number of simulations in this study:

(9) $$n={\left(\frac{z\sigma }{E}\right)}^{2}$$

where n is the minimum number of samples, σ is standard deviation, E is a margin of error, and z is the confidence coefficient for a given confidence level. 

Table 4 presents the calculation results for the minimum number of simulations. For total fracture work and peak force, the minimum numbers of simulations to reach a 95% confidence level are 5.449 and 2.162, respectively. Therefore, six models were finally selected for modeling the fracture behavior of AC in this study.

## 5. Results and Discussion

Following the verification of the numerical model against the laboratory data, a comprehensive parametric study was conducted to evaluate the fracture performance of the asphalt concrete. This section details how varying the aggregate gradation, volume content, and particle shape influences the internal stress state and macroscopic crack propagation within the mixture.

### 5.1. Effects of Gradation and Aggregate Volume Content

**Figure 6:**
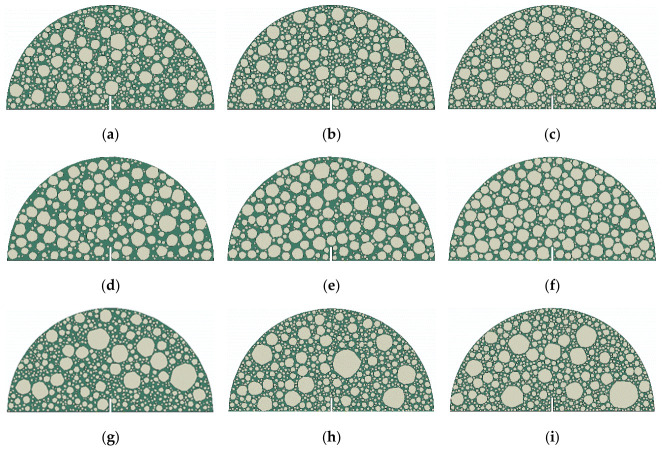
SCB models with different aggregate gradations and volume contents: (**a**) WC10 (0.55); (**b**) WC10 (0.60); (**c**) WC10 (0.65); (**d**) SMA10 (0.55); (**e**) SMA10 (0.60); (**f**) SMA10 (0.65;) (**g**) WC20 (0.55); (**h**) WC20 (0.60); (**i**) WC20 (0.65).

To systematically evaluate the effect of aggregate gradation on fracture properties, three distinct AC mixtures were investigated: WC10, WC20, and SMA10. WC10 and WC20 serve as dense-graded mixtures with nominal maximum aggregate sizes (NMASs) of 10 mm and 20 mm, respectively. SMA10 is a gap-graded stone mastic asphalt mixture with an NMAS of 10 mm. Based on the gradations of aggregate larger than 1.18 mm (aggregate smaller than 1.18 mm was considered in the FAM), the compositions of each mixture were determined as presented in Table 5. Three typical volume ratios for aggregates larger than 1.18 mm were evaluated: 0.55, 0.60, and 0.65. By combining these three gradations with the three volume contents, a total of 54 (3 × 3 × 6) microstructural models, as shown in Figure 6, were developed and simulated to ensure statistical reliability.

**Figure 7:**
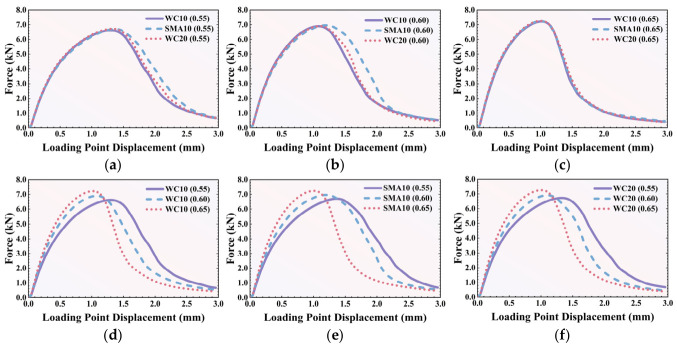
Average force–displacement curves of simulations: (**a**) 0.55 volume content; (**b**) 0.60 volume content; (**c**) 0.65 volume content; (**d**) WC10; (**e**) SMA10; (**f**) WC20.

**Figure 8:**
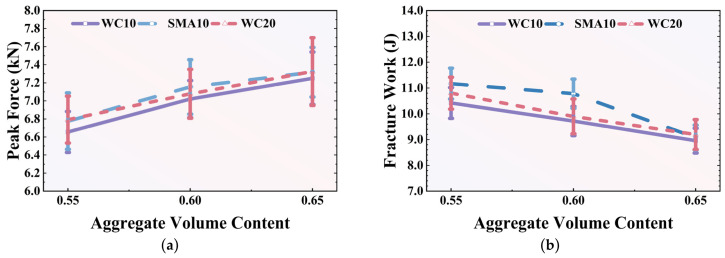
Effects of aggregate volume content on peak forces and fracture work: (**a**) peak force vs. aggregate volume content; (**b**) fracture work vs. aggregate volume content.

The simulated average force–displacement curves for these models are presented in Figure 7. The corresponding statistical analysis of the peak force and total fracture work is detailed in Table 5 and Figure 8. The simulations reveal that aggregate gradation exerts a highly significant influence on crack propagation behavior after the peak force is reached; however, this influence diminishes as the total aggregate volume content increases.

As Figure 7a,b show, at the low (0.55) and medium (0.60) volume contents of aggregate, the ranking of the force loss rate is: SMA10 < WC20 < WC10. From Figure 8, it can also be observed that with the same aggregate volume content, SMA10 showed the highest peak load and fracture work compared with the other two mixtures. Since large aggregate particles have a higher probability of blocking crack propagation than small aggregates, a higher concentration of large-sized particles corresponds to better cracking resistance. As Table 4 shows, the majority of the aggregates in SMA10 are larger than 5 mm, which likely leads to the lowest force loss rate. Due to the existence of some larger aggregates (greater than 10 mm), WC20 showed better cracking resistance than WC10. However, this trend disappeared when the aggregate volume content reached 0.65. This is likely because, at a high aggregate volume content, the overall blocking effect of the aggregate particles is uniformly intensified, and the individual effect of the coarse aggregate gradation on crack propagation becomes less significant.

The effect of aggregate volume content on fracture performance is illustrated in Figure 7d–f. Regardless of the gradation, both the reaction force increasing rate before the peak force and the loss rate after the peak force increase when the aggregate volume content increases from 0.55 to 0.65. In addition, the peak force also increases with the increase in aggregate volume content for all mixtures, which can also be observed in Figure 8a. These characteristics are associated with the blocking effect of the aggregate: a higher aggregate volume content leads to a stronger structural blocking effect. However, when the aggregate volume content increases, the overall fracture work decreases (see Figure 8b) and the mixture exhibits worse deformation ability, characterized by smaller loading displacements and a faster loss of loading capacity. Therefore, it can be concluded that a high aggregate volume content can postpone crack initiation by providing high initial strength, while simultaneously deteriorating the long-term deformation capability of the AC.

From a practical engineering perspective, these findings highlight crucial considerations for pavement design. They demonstrate that mix designers must avoid excessively high coarse aggregate contents that lead to overly brittle mixtures. A meticulously balanced volume ratio is crucial to ensure the pavement maintains sufficient flexibility and energy dissipation capacity to resist fatigue cracking under repeated traffic loads [15,46]. Furthermore, the superior post-peak performance of SMA10 provides computational validation for prioritizing gap-graded mixtures in high-stress pavement sections.

### 5.2. Effects of Aggregate Shape

**Figure 9:**
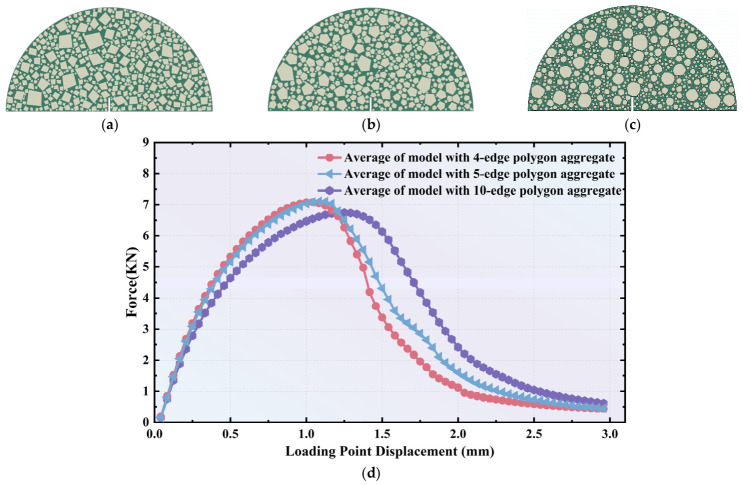
Effects of aggregate shape on the fracture behavior of AC: (**a**) model with 4-edge polygon; (**b**) model with 5-edge polygon; (**c**) model with 10-edge polygon; (**d**) force–displacement curves for model with varying aggregate shape.

To evaluate the influence of aggregate shape on the fracture performance of the asphalt mixture, polygonal aggregate particles represented by 4-edge (quadrilateral), 5-edge (pentagonal), and 10-edge geometries were developed for the WC10 mixture, as illustrated in Figure 9a–c. As the number of edges increases, the aggregate particles become increasingly round. For each particle shape category, 6 random models were generated, resulting in a total of 18 microstructural models designed to simulate fracture behavior while accounting for the effect of aggregate morphology.

The predicted average force–displacement curves for the mixtures with varying aggregate shapes are presented in Figure 9d. It can be observed that as the number of particle edges increases, the curve shifts to the right, exhibiting decreased tangent stiffness, a delayed peak force, and a slower reduction in stress following the peak force. This behavioral trend is opposite to the effects observed when increasing the aggregate volume content.

For polygonal aggregates with fewer edges, the increased angularity and sharper geometric variations make it more difficult for propagating cracks to bypass the particles. Consequently, the system requires a greater accumulation of energy to overcome this blocking effect, which leads to a higher peak force and a more rapid reduction in force after the peak stress is reached. Conversely, as the number of edges increases and the particles become more rounded, it becomes more feasible for cracks to propagate smoothly along the aggregate periphery. This results in a lower peak force and a more gradual reduction in force post-peak. Therefore, it can be concluded that incorporating particles with higher angularity is superior for the mixture, as they more effectively block crack propagation.

It should be noted that aggregate morphology is commonly described across multiple length scales by its form, angularity, roughness, and texture [53,54]. However, evidence consistently shows that macro-scale form is the dominant factor influencing crack initiation and propagation trajectories in asphalt mixtures. Macro-scale form characteristics—such as sphericity, flatness, elongation, and overall angularity—govern particle interlock, load transfer, and the mechanical skeleton of the mixture, making them the primary drivers of cracking behavior [55,56,57]. By contrast, micro-scale surface features such as roughness and texture mainly affect binder adhesion and certain secondary behaviors (e.g., moisture damage resistance) rather than the fundamental mechanisms that control macroscopic crack formation and growth [58,59,60]. Although these micro-properties contribute to interfacial bonding, their influence on the overall fracture work is generally minor compared to the structural blocking role played by aggregate form, especially when gradation and binder type are held constant. To address this limitation, our future studies will explicitly evaluate these microscale effects utilizing nanoindentation and Scanning Electron Microscopy (SEM) to characterize the microstructure and micromechanics of the asphalt concrete interface during cracking.

### 5.3. The Reliability of 2D Mesoscale Modeling

Three-dimensional (3D) mesoscale models provide recognized benefits for simulating the fracture behavior of asphalt concrete. Published studies report that 3D models more realistically represent complex spatial aggregate morphology, enhancing the accuracy of stress transfer, interlocking, and tortuous fracture surface representations [61,62,63]. However, high-fidelity 3D modeling demands substantial computational resources, strict material parameter identification, and complex mesh generation [27,64]. These limitations make 3D models less practical for simulating fracture tests such as the SCB configuration, where in-plane crack propagation dominates and out-of-plane effects contribute minimally to the primary failure mechanism.

In contrast, 2D mesoscale models provide clear advantages in efficiency, reproducibility, and analytical feasibility. Several studies demonstrate that 2D FEM, DEM, and peridynamic simulations reliably capture SCB crack initiation, crack path evolution, and fracture energy when random aggregate structures and cohesive zone interfaces are properly incorporated [27,65,66,67] Because the SCB test is fundamentally dominated by in-plane crack initiation and propagation, the out-of-plane mechanisms captured in 3D models contribute minimally to the macroscopic fracture process [27,68]. Crucially, the lower computational demand of 2D modeling enables the use of multiple random mesoscale realizations [69]. As demonstrated in this study, generating 50 distinct models allows researchers to quantify the influence of aggregate distribution, gradation, and shape with rigorous statistical robustness, effectively eliminating the analytical bias inherent to single-section 2D models.

The reliability of the 2D mesoscale SCB fracture model used in this study is strongly supported by the previous literature, which confirms that 2D models achieve accurate and experimentally supported outcomes when randomness and cohesive behavior are integrated. Prior works show that 2D fracture models employing polygonal aggregate geometries reproduce experimental observations of crack deflection, interfacial debonding, and low-temperature fracture responses with high consistency [27,32,70]. Peridynamic and CZM-based 2D approaches have similarly proven effective in simulating micro-crack initiation and propagation patterns under fracture loading, closely matching observed behavior in SCB and beam-bending experiments [67].

In this study, the mesoscale heterogeneous models generated by the random aggregate distribution algorithm and the cohesive zone model were utilized to evaluate the effects of varying shapes, gradations, and aggregate volume fractions. Evidence from mesoscale simulations shows that polygonal aggregate geometries, including the 4-, 5-, and 10-edge aggregate shapes used in the present work, successfully replicate the aggregate interlock, crack deflection, and tortuosity effects observed in experimental fracture tests. Moreover, cohesive zone models applied in 2D analyses reliably capture the low-temperature micro-crack initiation, crack coalescence, and interface debonding phenomena central to SCB fracture. These findings strongly validate the modeling strategy adopted in this study, which integrates polygonal aggregate structures and cohesive interfaces to accurately examine fracture performance at 10 °C.

## 6. Summary and Findings

In this study, the fracture performance of asphalt concrete (AC) was systematically analyzed through numerical modeling, specifically by coupling a random aggregate distribution algorithm with the cohesive zone model (CZM). Furthermore, the validated model was utilized to investigate the effects of aggregate gradation, volume content, and particle shape. Based on the simulation outcomes, the main findings and their practical engineering implications are summarized as follows:

- The proposed 2D numerical framework effectively and efficiently models the macroscopic fracture mechanics of heterogeneous AC. By generating 50 distinct random microstructural realizations, the study captured the inherent stochastic variation caused by different spatial aggregate distributions, ensuring that the predicted peak forces and fracture work achieved rigorous statistical reliability.
- Mixtures containing a higher percentage of large-sized coarse aggregates, specifically the gap-graded SMA10 and dense-graded WC20, demonstrated superior post-peak cracking resistance. The physical presence of larger macro-particles creates a stronger structural blocking effect, significantly increasing the energy required for a crack to bypass the aggregate phase. In engineering practice, this provides computational validation for prioritizing gap-graded mixtures in high-stress pavement sections.
- Increasing the coarse aggregate volume content (from 0.55 to 0.65) results in a higher peak force required to initiate cracking; however, it simultaneously leads to a more rapid deterioration in post-peak load-bearing capacity and a reduction in total fracture work. This indicates a critical loss in the composite’s overall deformation capacity. Mix designers must carefully balance volume ratios and avoid excessively high coarse aggregate contents to prevent overly brittle pavements that fail rapidly under repeated traffic loads.
- Higher aggregate angularity (represented by polygons with fewer edges) more effectively blocks crack propagation within the mixture by forcing cracks into highly tortuous paths. Conversely, more rounded aggregates allow cracks to easily bypass the particles and propagate smoothly through the asphalt matrix. This provides rigorous mechanical justification for material specifications mandating the use of highly angular crushed stone over rounded river gravel to maximize pavement fatigue resistance.

Overall, this research verifies the effectiveness and reliability of coupling a random aggregate distribution algorithm with the CZM to evaluate the fracture performance of AC. However, certain limitations in the current modeling approach should be noted. To ensure computational efficiency and allow for large-scale statistical validation, the simulations were restricted to 2D models, which may not fully capture the complex 3D spatial interactions and out-of-plane mechanisms of actual crack propagation. Additionally, the aggregate particles were mathematically simplified as 2D polygons. While these are practical, established, and highly effective engineering simplifications, they naturally deviate from real-world 3D fracturing behaviors. In future studies, this methodological framework will be extended to address these dimensional constraints, and microscale laboratory tests (e.g., nanoindentation and SEM) will be integrated to further evaluate the multi-scale mechanics of asphalt interface cracking.

## Figures and Tables

**Table 1:** Gradation and compositions of WC10 and FAM.

Sieve Size (mm)	WC10			FAM	
	Passing Percentage (%)	Mass Composition (%)	Volume Composition (%)	Passing Percentage (%)	Mass Composition (%)
14	100	5.6	5.2	100	-
10	94	24.4	22.3	100	-
5	68	16	14.6	100	-
2.36	51	16	14.6	100	-
1.18	34	10.3	43.3	100	33.2
0.6	23	8.5		66.8	27.2
0.3	14	4.7		39.6	15.1
0.15	9	2.8		24.5	9.1
0.075	6	5.6		15.5	14.9
Binder	-	6		-	15.5

**Table 2:** Prony series model parameters for FAM at 10 °C.

Series No.	$\tau_{i}$ (s)	$E_{i}$ (MPa)
1	5.626 × 10^−5^	4.09 × 10^3^
2	2.387 × 10^−4^	1.03 × 10^3^
3	1.012 × 10^−3^	2.74 × 10^3^
4	4.294 × 10^−3^	1.78 × 10^3^
5	1.822 × 10^−2^	1.92 × 10^3^
6	7.727 × 10^−2^	1.50 × 10^3^
7	3.278 × 10^−1^	1.13 × 10^3^
8	1.390	5.68 × 10^2^
9	5.898	1.94 × 10^2^
10	2.502 × 10	6.10 × 10
11	1.061 × 10^2^	1.45 × 10
12	4.502 × 10^2^	5.91
$E_{0}=15025.78$ MPa		

**Table 3:** T-test results.

	Total Fracture Work (J)		Peak Force (kN)	
	Simulation	Experiment	Simulation	Experiment
Mean	10.249	10.188	6.800	6.834
Variance	0.365		0.064	
Observations	50		50	
t Stat	0.267		0.347	
t Critical Two-Tailed	2.009		2.009	
Null Hypothesis (H0) (Experiment = Simulation)	Accepted (t Stat < t Critical)		Accepted (t Stat < t Critical)	

**Table 4:** Calculation of the minimum number of models.

	Total Fracture Work (J)	Peak Force (kN)
Number of Samples	50	50
Average	10.249	6.800
Standard Deviation	0.610	0.255
Individual Min. Number of Samples	5.449	2.162
Final Min. Number of Samples	6	

**Table 5:** Gradations and aggregate compositions.

Sieve Size (mm)	WC10		SMA10		WC20	
	Percent Passing (%)	Composition (%)	Percent Passing (%)	Compositions (%)	Percent Passing (%)	Composition (%)
28	-	-	-	-	100.0	-
20	-	-	-	-	94.0	6.0
14	100.0	-	100.0	-	82.0	12.0
10	90.9	9.1	92.5	7.5	72.9	9.1
5	51.5	39.5	19.1	73.4	51.9	21.0
2.36	25.8	25.8	7.9	11.2	23.3	28.6
1.18	0.0	25.8	0.0	7.9	0.0	23.3

## Data Availability

The original contributions presented in this study are included in the article. Further inquiries can be directed to the corresponding authors.

## Abbreviations

The following abbreviations are used in this manuscript:

CZM	Cohesive Zone Model
FAM	Fine Aggregate Matrix
SCB	Semi-circular Bending
AC	Asphalt Concrete
DIP	Digital Image Processing
WLF	Williams–Landel–Ferry
FE	Finite Element
MHS	Modified Huet–Sayegh
NMAS	Nominal Maximum Aggregate Size
DSR	Dynamic Shear Rheometer
SGC	Superpave Gyratory Compactor

## References

[1] Tan Z., Li H., Leng Z., Jelagin D., Cao P., Du C., Yin B. (2024). Constitutive modelling and systematic evaluation of asphalt concrete’s viscoelastic tension-compression asymmetry effect on pavement performance. Int. J. Pavement Eng..

[2] Tan Z., Leng Z., Li H., Ashish P.K., Cai X., Cao P., Sreeram A. (2024). Quantitative analysis of asphalt concrete’s tension-compression asymmetry effects on pavement response through 3D numerical modeling with dual viscoelastic model. Constr. Build. Mater..

[3] Tan Z., Leng Z., Gong M., Cao P., Xu C. (2026). Thermo-mechanical modeling of pavement’s response considering asphalt concrete’s tension-compression asymmetry. Comput.-Aided Civ. Infrastruct. Eng..

[4] Song W., Wu H., Yan W. (2024). Size effect analysis of mode I fracture performance of hot mix asphalt. Eng. Fract. Mech..

[5] Zhao Y., Hasan M.R.M., Zhang K., You L., Jamshidi A., Jiang J. (2025). XGBoost-based intelligent framework for asphalt pavement skid resistance assessment under different variables. Smart Constr..

[6] Malihi S., Potseluyko L., Mathew A., Alavi H., Kumar Reja V., Pan Y., Binni L., Wang G., Wang X., Brilakis I. (2024). Review of multimodal data and their applications for road maintenance. Smart Constr..

[7] Tan X., Yang J., Zhuang H., Xu J., Gao J., Wan T., Zhang J. (2025). Exploring the physical hardening characteristics of asphalt mortars and mixtures based on gradient particle size. Constr. Build. Mater..

[8] Sun Y., Du C., Gong H., Li Y., Chen J. (2020). Effect of temperature field on damage initiation in asphalt pavement: A microstructure-based multiscale finite element method. Mech. Mater..

[9] Tan Z., Yang B., Leng Z., Jelagin D., Cao P., Li R., Zou F. (2023). Multiscale characterization and modeling of aggregate contact effects on asphalt concrete’s tension–compression asymmetry. Mater. Des..

[10] Tan Z., Leng Z., Jiang J., Cao P., Jelagin D., Li G., Sreeram A. (2022). Numerical study of the aggregate contact effect on the complex modulus of asphalt concrete. Mater. Des..

[11] Song W., Deng Z., Wu H., Xu Z. (2023). Cohesive zone modeling of I–II mixed mode fracture behaviors of hot mix asphalt based on the semi-circular bending test. Theor. Appl. Fract. Mech..

[12] Castillo D., Caro S., Darabi M., Masad E. (2018). Influence of aggregate morphology on the mechanical performance of asphalt mixtures. Road Mater. Pavement Des..

[13] Ameri M., Aliha M.R.M., Ebrahimzadeh Shiraz M., Tamizi T. (2025). Investigating the effect of specimens, materials, and environmental factors on fracture properties of asphalt mixtures: A literature review. Innov. Infrastruct. Solut..

[14] Tan Z., Guo Y., Hu G., Chen R., Wang Y., Yin B., Leng Z. (2025). Upcycling waste wind turbine blades into fiber-reinforced asphalt mortar: A chemical recycling approach and performance assessment. Constr. Build. Mater..

[15] Li X., Shi L., Liao W., Wang Y., Nie W. (2024). Study on the influence of coarse aggregate morphology on the meso-mechanical properties of asphalt mixtures using discrete element method. Constr. Build. Mater..

[16] Yan Y., Zhang H., Bekoe M., Allen C., Zhou J., Roque R. (2023). Effects of asphalt binder type, aggregate type, and gradation characteristics on fracture properties and performance of asphalt mixtures at intermediate temperatures. Constr. Build. Mater..

[17] Huang G., Chen Z., Wang S., Hu D., Zhang J., Pei J. (2024). Investigation of fracture failure and water damage behavior of asphalt mixtures and their correlation with asphalt-aggregate bonding performance. Constr. Build. Mater..

[18] Meng Y., Chen J., Kong W., Wang Z., Lu Y., Chen P. (2024). Research on the influence of parameters on the fracture performance of the large stone asphalt mixture based on the semi-circular bending test. Constr. Build. Mater..

[19] Tan Z., Leng Z., Jelagin D., Cao P., Jiang J., Kumar Ashish P., Zou F. (2023). Numerical modeling of the mechanical response of asphalt concrete in tension and compression. Mech. Mater..

[20] Espinosa L., Wills J., Caro S., Braham A. (2019). Influence of the morphology of the cracking zone on the fracture energy of HMA materials. Mater. Struct. Mater. Constr..

[21] Eghbali M.R., Tafti M., Aliha M.R.M. (2026). The Effect of Aggregate Gradation on the Fracture Resistance of Asphalt Mixtures Subjected to Freeze–Thaw Cycles. Fatigue Fract. Eng. Mater. Struct..

[22] Asghar M.F., Khattak M.J., Olayinka A. (2022). Evaluation of fracture performance of Polyvinyl Alcohol fiber reinforced hot mix asphalt. Constr. Build. Mater..

[23] Soleimani Golsefidi S., Ali Sahaf S. (2022). Effect of reclaimed asphalt pavement (RAP) on fracture properties of stone matrix asphalt (SMA) at low temperature. Constr. Build. Mater..

[24] Wang S., Cao H., Chen T., Ke W., Bo W. (2023). Research on the Fracture Characteristics of Asphalt Mixtures in High Altitude and Cold Regions with Large Temperature Differences. Coatings.

[25] Wang H., Cheng Y., Liang J., Zhao W., Li A. (2024). Evaluating the fracture characterization of asphalt mixtures under freeze-thaw damage based on full-field measurements. Measurement.

[26] Wei H., Li J., Wang F., Zheng J., Tao Y., Zhang Y. (2022). Numerical investigation on fracture evolution of asphalt mixture compared with acoustic emission. Int. J. Pavement Eng..

[27] Zhang H., Ding H., Rahman A. (2022). Effect of Asphalt Mortar Viscoelasticity on Microstructural Fracture Behavior of Asphalt Mixture Based on Cohesive Zone Model. J. Mater. Civ. Eng..

[28] Zhang Z., Song X., Liu Y., Wu D., Song C. (2017). Three-dimensional mesoscale modelling of concrete composites by using random walking algorithm. Compos. Sci. Technol..

[29] Xie L., Pang B., Cao P., Wang J., Tan Z. (2026). Mesoscale Steady-State Dynamics Modeling and Parametric Analysis of the Viscoelastic Response of Asphalt-Bonded Calcareous Sand. Materials.

[30] Zeng Z., Underwood B.S., Kim Y.R. (2024). A state-of-the-art review of asphalt mixture fracture models to address pavement reflective cracking. Constr. Build. Mater..

[31] Talebi H., Bahrami B., Ahmadian H., Nejati M., Ayatollahi M.R. (2024). An investigation of machine learning algorithms for estimating fracture toughness of asphalt mixtures. Constr. Build. Mater..

[32] Gao L., Zhou Y., Jiang J., Yang Y., Kong H. (2024). Mix-mode fracture behavior in asphalt concrete: Asymmetric semi-circular bending testing and random aggregate generation-based modelling. Constr. Build. Mater..

[33] Gu X., Xu X., Zhang Q., Sun L., Zhou Z. (2024). Study on the correlation between spatial variability of asphalt mixture material parameters and fracture performance. Case Stud. Constr. Mater..

[34] Dave E.V., Behnia B. (2018). Cohesive zone fracture modelling of asphalt pavements with applications to design of high-performance asphalt overlays. Int. J. Pavement Eng..

[35] Rodrigues J.A., Teixeira J.E.S.L., Kim Y.R., Little D.N., Souza F.V. (2019). Crack modeling of bituminous materials using extrinsic nonlinear viscoelastic cohesive zone (NVCZ) model. Constr. Build. Mater..

[36] Kim Y.R., Aragão F.T.S., Allen D.H., Little D.N. (2010). Damage modeling of bituminous mixtures considering mixture microstructure, viscoelasticity, and cohesive zone fracture. Can. J. Civ. Eng..

[37] Bekele A., Balieu R., Jelagin D., Ryden N., Gudmarsson A. (2021). Micro-mechanical modelling of low temperature-induced micro-damage initiation in asphalt concrete based on cohesive zone model. Constr. Build. Mater..

[38] Yazdipanah F., Bastola N.R., Faxina A.L., Lutif Teixeira J.E.S. (2026). Numerical predictions of cracking evolution on RAP-recycled asphalt mixtures using viscoelastic cohesive zone model with Gaussian damage function. Constr. Build. Mater..

[39] Kim Y.-R., de Freitas F.A.C., Jung J.S., Sim Y. (2015). Characterization of bitumen fracture using tensile tests incorporated with viscoelastic cohesive zone model. Constr. Build. Mater..

[40] Anderson D.A., Christensen D.W., Bahia H. (1991). Physical Properties of Asphalt Cement and the Development of Performance-related Specifications. Proceedings of the Asphalt Paving Technology; Proceedings of the Technical Sessions.

[41] Tan Z., Li H., Leng Z., Yin B., Li D., Zou F., Cao P. (2024). Fatigue performance analysis of fine aggregate matrix using a newly designed experimental strategy and viscoelastic continuum damage theory. Mater. Struct..

[42] Li H., Tan Z., Li R., Luo X., Zhang Y., Leng Z. (2024). Mechanistic modeling of fatigue crack growth in asphalt fine aggregate matrix under torsional shear cyclic load. Int. J. Fatigue.

[43] Woldekidan M.F., Huurman M., Pronk A.C. (2012). A modified HS model: Numerical applications in modeling the response of bituminous materials. Finite Elem. Anal. Des..

[44] Kollmann J., Lu G., Liu P., Xing Q., Wang D., Oeser M., Leischner S. (2019). Parameter optimisation of a 2D finite element model to investigate the microstructural fracture behaviour of asphalt mixtures. Theor. Appl. Fract. Mech..

[45] Baek J. (2010). Modeling Reflective Cracking Development in Hot-Mix Asphalt OVERLAYS and Quantification of Control Techniques.

[46] Underwood B.S., Kim Y.R. (2013). Effect of volumetric factors on the mechanical behavior of asphalt fine aggregate matrix and the relationship to asphalt mixture properties. Constr. Build. Mater..

[47] Huurman R.M., Mo L., Woldekidan M.F. (2010). Unravelling Porous Asphalt Concrete towards a Mechanistic Material Design Tool. Road Mater. Pavement Des..

[48] Zhang X., Gu X., Lv J., Zhu Z., Zou X. (2017). Numerical analysis of the rheological behaviors of basalt fiber reinforced asphalt mortar using ABAQUS. Constr. Build. Mater..

[49] Leng Z., Tan Z., Cao P., Zhang Y. (2021). An efficient model for predicting the dynamic performance of fine aggregate matrix. Comput.-Aided Civ. Infrastruct. Eng..

[50] Kim Y.-R. (2011). Cohesive zone model to predict fracture in bituminous materials and asphaltic pavements: State-of-the-art review. Int. J. Pavement Eng..

[51] Rami K.Z., Amelian S., Kim Y.-R., You T., Little D.N. (2017). Modeling the 3D fracture-associated behavior of viscoelastic asphalt mixtures using 2D microstructures. Eng. Fract. Mech..

[52] Guo F.-q., Zhang H., Yang Z.-j., Huang Y.-j., Withers P.J. (2023). A spherical harmonic-random field coupled method for efficient reconstruction of CT-image based 3D aggregates with controllable multiscale morphology. Comput. Methods Appl. Mech. Eng..

[53] Tan Z., Guo F.-q., Yu H., Cao P., Leng Z., Xu C. (2026). Virtual generation and quantitative characterization of 3D aggregate skeletons in asphalt mixtures. Powder Technol..

[54] Wang L., Shen A., Yao J. (2020). Effect of different coarse aggregate surface morphologies on cement emulsified asphalt adhesion. Constr. Build. Mater..

[55] Feng D. (2025). Multi-scale algorithm for controllable virtual aggregate generation in mesoscale modeling. Int. J. Mech. Sci..

[56] Gao J., Wang H., Bu Y., You Z., Hasan M.R.M., Irfan M. (2018). Effects of coarse aggregate angularity on the microstructure of asphalt mixture. Constr. Build. Mater..

[57] Liu H., Yan Z., Wang F., Bian W., Tang Y., Zhang J., Jiang W. (2025). Quantitative analysis of morphological features of recycled asphalt pavement and natural coarse aggregates using aggregate image measurement system. Case Stud. Constr. Mater..

[58] Darshan N., Kataware A.V., Suryawanshi S. (2026). Macro-micro-nano scale investigation of moisture resistant performance of warm asphalt mixes prepared with different asphalt binder and aggregate types. Constr. Build. Mater..

[59] Jin C., Zou F., Yang X., Liu K., Liu P., Oeser M. (2020). Three-dimensional quantification and classification approach for angularity and surface texture based on surface triangulation of reconstructed aggregates. Constr. Build. Mater..

[60] Ge H., Quezada J.C., Le Houerou V., Chazallon C. (2021). Three-dimensional simulation of asphalt mixture incorporating aggregate size and morphology distribution based on contact dynamics method. Constr. Build. Mater..

[61] Han D., Xi Y., Xie Y., Li Z., Zhao Y. (2023). 3D Virtual reconstruction of asphalt mixture microstructure based on rigid body dynamic simulation. Int. J. Pavement Eng..

[62] Tan Z., Guo F.-q., Leng Z., Yang Z.-J., Cao P. (2024). A novel strategy for generating mesoscale asphalt concrete model with controllable aggregate morphology and packing structure. Comput. Struct..

[63] Chen Y., Wan C., Alae M., Xiao F. (2025). Advances in simulation parameters and methods for three-dimensional mesoscopic model of asphalt mixture. Front. Struct. Civ. Eng..

[64] Zhao Y., Jiang J., Zhou L., Ni F. (2022). Improving the calculation accuracy of FEM for asphalt mixtures in simulation of SCB test considering the mesostructure characteristics. Int. J. Pavement Eng..

[65] Nian T., Ge J., Li P., Wang M., Mao Y. (2021). Improved discrete element numerical simulation and experiment on low-temperature anti-cracking performance of asphalt mixture based on PFC2D. Constr. Build. Mater..

[66] Zhao Y., Zhang Y., Jiang J. (2021). Application and improvement of discrete finite-element method for mesoscale fracture analysis of asphalt mixtures. J. Transp. Eng. Part B Pavements.

[67] Ruan L., Luo R., Zhang D., Wang B. (2021). Numerical simulation of crack paths in asphalt mixture using ordinary state-based peridynamics. Mater. Struct..

[68] Zhu T., Chen Z., Cao J., Wang Z., Hao J., Zhou Z. (2025). Crack resistance of cemented waste rock tailings backfill under splitting tensile load: Experimental and numerical investigations. J. Build. Eng..

[69] Zhao Y., Jiang J., Zhou L., Dai Y., Ni F. (2021). Meso-structure image pre-selection method for two-dimensional finite element modeling in beam bending simulation of asphalt mixture. Constr. Build. Mater..

[70] Chen A., Airey G.D., Thom N., Li Y., Wan L. (2022). Simulation of micro-crack initiation and propagation under repeated load in asphalt concrete using zero-thickness cohesive elements. Constr. Build. Mater..

